# Clinical Outcomes of Hypoglossal Nerve Stimulation Versus Continuous Positive Airway Pressure in Obstructive Sleep Apnea

**DOI:** 10.1002/oto2.70240

**Published:** 2026-04-20

**Authors:** Amala Nayak, Iman Adibi, Alyssa Calder, Arman Saeedi, Aaron Tucker, Ryan Nord

**Affiliations:** ^1^ Department of Otolaryngology–Head and Neck Surgery Virginia Commonwealth University School of Medicine Richmond PO BOX Virginia USA

**Keywords:** continuous positive airway pressure, hypoglossal nerve stimulation, obstructive sleep apnea

## Abstract

**Objectives:**

Obstructive sleep apnea (OSA) is linked to cardiovascular, metabolic, and neuropsychiatric morbidity. Continuous positive airway pressure (CPAP) remains first‐line therapy, but poor adherence limits effectiveness. Hypoglossal nerve stimulation (HNS) is an emerging alternative for CPAP‐intolerant patients. This study compared clinical outcomes between HNS and CPAP in OSA patients.

**Study Design:**

Retrospective Cohort Study.

**Setting:**

TriNetX Research Network.

**Methods:**

We identified adults with OSA who underwent HNS implantation or initiated CPAP therapy, with 2 years of follow‐up. Propensity score matching (n = 3525 per group) balanced baseline demographics and comorbidities. Acute cardiovascular, respiratory, and metabolic outcomes were assessed from 30 days posttreatment to 2 years. A similar analysis was performed to compare health outcomes of HNS and uvulopalatopharyngoplasty (UPPP) surgery without future CPAP use.

**Results:**

The HNS cohort had significantly lower odds of stroke (odds ratio [OR] 0.626, *P* = .0085), myocardial infarction (OR 0.612, *P* < .0108), atrial fibrillation/flutter (OR 0.594, *P* < .0001), hypertensive crisis (OR 0.456, *P* < .0274), pulmonary embolism (OR 0.209, *P* < .0001), ventricular tachycardia (OR 0.349, *P* = .0001), COPD exacerbation (OR 0.270, *P* < .0001), acute kidney injury (OR 0.283, *P* < .0001), ED visit (OR 0.457, *P* < .0001), hospitalization (OR 0.419, *P* < .0001), acute heart failure (OR 0.198, *P* < .0001), heart failure exacerbation (OR 0.221, *P* < .0001), acute respiratory failure (OR 0.172, *P* < .0001), and pneumonia (OR 0.255, *P* < .0001). Daytime sleepiness was more common in the HNS group (OR 2.019, *P* < .0001). HNS and UPPP cohorts displayed largely similar health outcomes.

**Conclusion:**

HNS may offer systemic benefits and reduce healthcare burden compared to CPAP. Future studies should incorporate adherence data and cost‐effective analyses to guide treatment.

Obstructive sleep apnea (OSA) is a chronic respiratory disorder characterized by recurrent episodes of upper airway obstruction during sleep, leading to intermittent hypoxia, hypercapnia, and frequent arousals, disrupting normal sleep architecture.^1^ The pathophysiology of OSA is multifactorial, involving upper airway narrowing and impaired neuromuscular control of airway dilator muscles.^2^ Untreated OSA contributes to substantial morbidity and mortality including increased risk of hypertension, cardiovascular diseases, metabolic dysfunction, neurocognitive impairments, and chronic mental health issues through mechanisms involving chronic sympathetic nervous system activation, systemic inflammation, oxidative stress, and endothelial dysfunction, all triggered by repetitive hypoxic and arousal events.^1^, ^2^

Continuous positive airway pressure (CPAP) therapy is the first‐line treatment for OSA patients.^3^ CPAP delivers pressurized air through a mask to maintain upper airway patency during sleep.^4^ CPAP restores sleep architecture, alleviates daytime sleepiness, improves quality of life, and has beneficial effects on certain cardiovascular risk factors, particularly with optimal adherence.^5^

Despite the high efficacy of CPAP, its effectiveness is hampered by suboptimal adherence.^5^ Reported adherence rates vary widely, with many patients using the device for fewer than the recommended 4 hours per night or discontinuing therapy altogether due to issues with tolerance.^5^, ^6^ Therefore, many nonadherent CPAP patients remain at risk for the long‐term sequelae of untreated OSA.^6^ This challenge has driven the search for alternative therapies.

Hypoglossal nerve stimulation (HNS) has emerged as an alternative therapy for patients with moderate to severe OSA who cannot tolerate or achieve benefit from CPAP.^7^ HNS systems, like the Inspire® device, are surgically implanted to deliver respiratory‐synchronized electrical stimulation to the hypoglossal nerve, selectively activating the genioglossus muscle leading to tongue protrusion and resultant retroglossal and retropalatal airway opening, which mitigates upper airway collapse during sleep.^7^ HNS is indicated for patients who meet specific anatomical and physiological criteria and have documented CPAP failure or intolerance.^8^

Other commonly performed surgical therapies such as uvulopalatopharyngoplasty (UPPP) have been done for decades to treat OSA.^9^ UPPP surgically corrects obstruction at the level of the oropharynx by partial resection of the soft palate, uvula, and tonsils, although it does not address the increased collapsibility of the upper airway in OSA patients.^10^

Both HNS and CPAP effectively reduce the AHI and improve daytime sleepiness and quality of life.^11^ Comparative studies suggest HNS may offer greater improvements in subjective outcomes like daytime sleepiness, potentially due to higher adherence rates.^11^ For cardiovascular outcomes such as myocardial infarction and stroke, CPAP has extensive evidence, with benefits strongly linked to adherence.^5^ Evidence for HNS in these areas is still emerging, with current hypotheses suggesting potential benefits driven by its favorable adherence profile; however, direct, long‐term comparative data are largely lacking.^11^ Similarly, data on renal outcomes for HNS are scarce. For pulmonary outcomes in OSA‐COPD overlap syndrome, CPAP remains the standard, as HNS does not affect lower airway disease.^12^, ^13^ Neuropsychiatric benefits, including improvements in depression and insomnia, are evident with both therapies.^14^, ^15^, ^16^, ^17^ Reductions in healthcare resources and hospitalizations are documented with adherent CPAP use; similar benefits are inferred but not directly proven for HNS.^18^

The primary purpose of this study was to compare a wide array of health outcomes spanning acute cardiovascular, renal, pulmonary, neuropsychiatric, and healthcare utilization domains between OSA patients treated with HNS versus CPAP. Factors such as device efficacy and patient adherence, combined as the concept of mean disease alleviation (MDA), appear to play an integral role in outcomes between these treatment modalities.^19^, ^20^

## Methods

We conducted a retrospective cohort study using the TriNetX Research Network, a federated database of de‐identified electronic health records from over 100 US healthcare organizations and more than 150 million patients. Patients with an OSA diagnosis were identified via ICD‐10 codes. TriNetX is exempt from human subject research regulations and does not require IRB approval.

**Figure 1:**
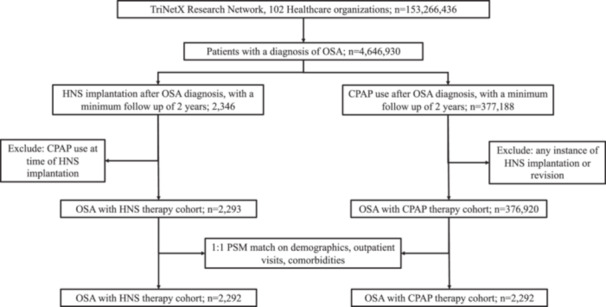
Flow diagram outlining inclusion, exclusion, and 1:1 propensity score matching of OSA patients receiving HNS versus CPAP therapy based on demographics, comorbidities, and outpatient visit frequency.

Two mutually exclusive cohorts were created: (1) patients who underwent HNS implantation after OSA diagnosis with at least 2 years of follow‐up and (2) patients who initiated CPAP therapy with similar follow‐up. CPAP initiation was defined as the first coded use after diagnosis. Patients were excluded from the HNS cohort if they were using CPAP at the time of implantation, and from the CPAP cohort if they had any record of HNS use. Cohort selection is summarized in Figure 1.

We performed 1:1 propensity score matching (PSM) between HNS and CPAP cohorts based on age at index, sex, race/ethnicity, BMI, outpatient visit frequency, tobacco/nicotine use, and comorbidities (eg, hypertension, diabetes, CAD, CKD, sinusitis). PSM was conducted using TriNetX's logistic regression algorithm, and matching was considered successful at a standardized mean difference <0.10.^21^, ^22^

Post‐PSM, we assessed adverse clinical outcomes occurring 30 days to 2 years after therapy initiation. A 30‐day washout period accounted for HNS activation time and excluded transient inpatient CPAP exposure. Given the delay in therapeutic titration of HNS, a 2‐year window was used to capture long‐term effects. Outcomes included stroke, MI, atrial fibrillation/flutter, aortic dissection, heart failure exacerbation, respiratory failure, hypertensive crisis, pulmonary embolism, pneumonia, ventricular arrhythmias, COPD exacerbation, suicide attempt, AKI, excessive daytime sleepiness (hypersomnolence/somnolence), ED visits, and hospitalizations. Odds ratios (OR) with 95% confidence intervals (CI) were calculated within TriNetX to compare HNS and CPAP groups. To assess whether observed outcome differences reflected baseline disparities (eg, better health or socioeconomic status in HNS patients) influenced by surgical candidacy, we conducted a parallel analysis comparing HNS and UPPP cohorts. ICD‐10 codes for cohort selection and outcomes are listed in Table 1.

**Table 1:** ICD‐10 and CPT Codes for TriNetX Query Cohort Building and Outcome Analysis

Cohort building	ICD‐10/CPT code
OSA	G47.33
HNS implantation	64582, 64583
CPAP use	5A09357, 5A09457, 5A09557, 94660
Outcomes	ICD‐10/CPT code
Stroke	I63
Myocardial infarction	I21
Atrial fibrillation or atrial flutter	I48
Hypertensive crisis	I16
Pulmonary embolism	I26
Ventricular tachycardia	I47.2
Chronic obstructive pulmonary disease exacerbation	J44.1
Suicide attempt	T14.91
Acute kidney injury	N17
Daytime sleepiness	R40.0, G47.10, G47.19
Emergency department visit	1013711, SNOMED 4525004
Hospitalization	1013699, 1013659, SNOMED 737481003, “Visit: Inpatient Encounter”
Aortic dissection	I71.00
Acute heart failure	I50.21, I50.41, I50.813, I50.811, I50.31, I50.21, I50.33, I50.23, I50.43, I50.41
Transient ischemia attack	G45
Heart failure exacerbation	I50.33, I50.23, I50.43
Acute respiratory failure	J96.0
Sudden cardiac death	I46
Pneumonia	J18
Ventricular fibrillation	I49.01, I49.0

## Results

A total of 383,658 patients undergoing CPAP therapy and 2293 patients treated with HNS for OSA were identified prior to PSM. Prior to matching, significant differences were observed between cohorts in sex, race/ethnicity, and multiple comorbidities (standardized difference [SMD] > 0.1) (Table 2). Patients in the HNS cohort had a higher proportion of males (65.0% vs 57.9%, SMD = 0.146), and were more likely to be white (85.4% vs 72.4%, SMD = 0.323). Comorbid conditions were also more prevalent in the CPAP group before matching, including hypertensive diseases (77.5% vs 57.2%, SMD = 0.444), diabetes mellitus (47.3% vs 21.4%, SMD = 0.567), chronic ischemic heart disease (36.0% vs 17.1%, SMD = 0.438), nicotine dependence (20.2% vs 11.9%, SMD = 0.225) and CKD (27.8% vs 9.2%, SMD = 0.494). The HNS cohort had a higher proportion of patients with BMI 20 to 29 (18.4% vs 4.4%, SMD = 0.452) and BMI 30 to 39 (25.2% vs 19.8%, SMD = 0.128). The CPAP cohort had a higher proportion of patients with BMI over 40 (23.4% vs 0.7%, SMD = 0.741).

**Table 2:** 1:1 PSM of HNS and CPAP Cohorts

	Before PSM			After PSM		
Characteristic name	HNS (n = 2293); n (%)	CPAP (n = 383,658); n (%)	SMD	HNS (n = 2292); n (%)	CPAP (n = 2292); n (%)	SMD
Age at index	62.1 ± 11.5	61.1 ± 15.9	0.073	62.1 ± 11.5	61.8 ± 13.3	0.020
White	1959 (85.4)	277,852 (72.4)	**0.323**	1958 (85.4)	1938 (84.6)	0.024
Male	1490 (65.0)	222,095 (57.9)	**0.146**	1489 (65.0)	1518 (66.2)	0.027
Female	763 (33.3)	158,895 (41.4)	**0.169**	763 (33.3)	732 (31.9)	0.029
Black or African American	90 (3.9)	66,469 (16.8)	**0.432**	90 (3.9)	92 (4.0)	0.005
Hispanic or Latino	86 (3.8)	21,742 (5.7)	0.091	86 (3.8)	93 (4.1)	0.016
Asian	42 (1.8)	7334 (1.9)	0.006	42 (1.8)	45 (2.0)	0.010
Hypertensive diseases	1312 (57.2)	297,464 (77.5)	**0.444**	1311 (57.2)	1347 (58.8)	0.032
Disorders of lipoprotein metabolism and other lipidemias	1297 (56.6)	239,902 (62.5)	**0.122**	1296 (56.5)	1311 (57.2)	0.013
Encounter for other special examination without complaint, suspected or reported diagnosis	1149 (50.1)	139,347 (36.3)	**0.281**	1148 (50.1)	1162 (50.7)	0.012
BMI 30‐39, adult	577 (25.2)	76,075 (19.8)	**0.128**	576 (25.1)	562 (24.5)	0.014
Encounter for general examination without complaint, suspected or reported diagnosis	579 (25.3)	97,725 (25.5)	0.005	578 (25.2)	553 (24.1)	0.025
Diabetes mellitus	491 (21.4)	181,591 (47.3)	**0.567**	491 (21.4)	481 (21.0)	0.011
Depression, unspecified	437 (19.1)	47,218 (12.3)	**0.186**	436 (19.0)	406 (17.7)	0.034
BMI 20‐29, adult	423 (18.4)	16,952 (4.4)	**0.452**	422 (18.4)	421 (18.4)	0.001
Chronic ischemic heart disease	391 (17.1)	137,928 (36.0)	**0.438**	391 (17.1)	352 (15.4)	0.046
Nicotine dependence	274 (11.9)	77,330 (20.2)	**0.225**	274 (12.0)	259 (11.3)	0.020
Chronic sinusitis	283 (12.3)	35,214 (9.2)	**0.102**	282 (12.3)	274 (12.0)	0.011
Atrial fibrillation and flutter	220 (9.6)	99,271 (25.9)	**0.436**	220 (9.6)	221 (9.6)	0.002
Other disorders of fluid, electrolyte and acid‐base balance	208 (9.1)	153,416 (40.0)	**0.770**	208 (9.1)	190 (8.3)	0.028
Chronic kidney disease	210 (9.2)	106,543 (27.8)	**0.494**	210 (9.2)	208 (9.1)	0.003
Other chronic obstructive pulmonary disease	158 (6.9)	110,883 (28.9)	**0.600**	158 (6.9)	164 (7.2)	0.010
Heart failure	126 (5.5)	138,253 (36.0)	**0.813**	126 (5.5)	114 (5.0)	0.024
Disorder of kidney and ureter, unspecified	108 (4.7)	40,291 (10.5)	**0.220**	108 (4.7)	93 (4.1)	0.032
Acute kidney failure	97 (4.2)	106,313 (27.7)	**0.677**	97 (4.2)	104 (4.5)	0.015
Tobacco use	82 (3.6)	23,534 (6.1)	0.119	82 (3.6)	65 (2.8)	0.042
Atherosclerosis	87 (3.8)	40,392 (10.5)	**0.263**	87 (3.8)	67 (2.9)	0.048
Cerebral infarction	85 (3.7)	32,571 (8.5)	**0.201**	85 (3.7)	74 (3.2)	0.026
Acute myocardial infarction	75 (3.3)	44,613 (11.6)	**0.322**	75 (3.3)	49 (2.1)	0.070
Respiratory failure, not elsewhere classified	48 (2.1)	114,171 (29.8)	**0.817**	48 (2.1)	55 (2.4)	0.021
Other pulmonary heart diseases	43 (1.9)	63,960 (16.7)	**0.528**	43 (1.9)	45 (2.0)	0.006
BMI 40 or greater, adult	17 (0.7)	89,622 (23.4)	**0.741**	17 (0.7)	24 (1.0)	0.032
Other acute ischemic heart diseases	14 (0.6)	13,280 (3.7)	**0.216**	14 (0.6)	10 (0.4)	0.024

Abbreviations: CPAP, continuous positive airway pressure; HNS, hypoglossal nerve stimulation; PSM, Propensity score matching; SMD, standardized mean difference.

Propensity score matching was done at a 1:1 ratio. Significant difference (bolded) was set at a standardized mean difference >0.10.

After PSM (n = 2292 per group), baseline characteristics were well‐balanced with no significant differences between cohorts (SMD < 0.10 for all variables). The mean age was 62.1 ± 11.5 in the HNS group and 61.8 ± 13.2 in the CPAP group (SMD = 0.020). Gender distribution and racial composition were comparable (SMD < 0.10). All comorbidities were similarly distributed between the cohorts after matching.

**Figure 2:**
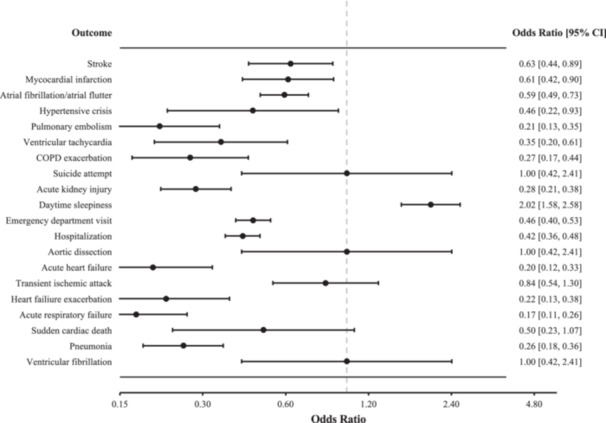
Two‐year odds ratios of adverse outcomes in OSA patients treated with HNS versus CPAP. Values below 1.0 indicate reduced risk with HNS therapy (n = 2292). CPAP, continuous positive airway pressure; HNS, hypoglossal nerve stimulation; OSA, obstructive sleep apnea.

Significant differences were observed in clinical outcomes between the HNS and CPAP groups following treatment (Figure 2, Table 3). At 2 years, patients in the HNS group had significantly lower rates of stroke (2.3% vs 3.6%, OR 0.63, 95% CI: 0.44‐0.89, *P* = .0085), myocardial infarction (1.9% vs 3.1%, OR 0.61, 95% CI: 0.42‐0.90, *P* = .0108), atrial fibrillation/flutter (7.4% vs 11.8%, OR 0.59, 95% CI: 0.49‐0.73, *P* < .0001), and hypertensive crisis (0.48% vs 1.05%, OR 0.46, 95% CI: 0.22‐0.93, *P* = .0274). HNS patients also had lower odds of pulmonary embolism (0.83% vs 3.84%, OR 0.21, 95% CI: 0.13‐0.35, *P* < .0001), ventricular tachycardia (0.74% vs 2.09%, OR 0.35, 95% CI: 0.20‐0.61, *P* = .0001), heart failure exacerbation (0.74% vs 3.27%, OR 0.22, 95% CI: 0.13‐0.38, *P* < .0001) and acute heart failure (0.83% vs 4.06%, OR 0.20, 95% CI: 0.12‐0.33, *P* < .0001). Similarly, lower rates of COPD exacerbation (0.92% vs 3.32%, OR 0.27, 95% CI: 0.17‐0.44, *P* < .0001), acute respiratory failure (1.13% vs 6.24%, OR 0.17, 95% CI: 0.11‐0.26, *P* < .0001), and acute kidney injury (2.62% vs 8.68%, OR 0.28, 95% CI: 0.21‐0.38 *P* < .0001), were observed in the HNS group. Rates of healthcare utilization were lower among HNS patients, including reduced ED visits (15.00% vs 27.88%, OR 0.46, 95% CI: 0.40‐0.53, *P* < .0001) and hospitalizations (15.23% vs 30.11%, OR 0.42, 95% CI: 0.36‐0.48, *P* < .0001). However, daytime sleepiness was more common in the HNS cohort (8.60% vs 4.45%, OR 2.02, 95% CI: 1.58‐2.58, *P* < .0001). Aortic dissection, transient ischemic attack, sudden cardiac death, ventricular fibrillation, and suicide attempt were not significant (*P* > .05).

**Table 3:** 30 Days to 2‐Year Outcomes of HNS Versus CPAP Therapy for OSA Patients

Outcome	HNS (n = 2292); n (%)	CPAP (n = 2292); n (%)	Odds ratio (95% CI)	*P*‐value
Stroke	52 (2.3)	82 (3.6)	0.626 (0.440‐0.890)	.0085
Myocardial infarction	44 (1.9)	71 (3.1)	0.612 (0.418‐0.896)	.0108
Atrial fibrillation/atrial flutter	169 (7.4)	271 (11.8)	0.594 (0.485‐0.726)	<.0001
Hypertensive crisis	11 (0.5)	24 (1.0)	0.456 (0.223‐0.933)	.0274
Pulmonary embolism	19 (0.8)	88 (3.8)	0.209 (0.127‐0.345)	<.0001
Ventricular tachycardia	17 (0.7)	48 (2.1)	0.349 (0.200‐0.609)	.0001
COPD exacerbation	21 (0.9)	76 (3.3)	0.270 (0.166‐0.439)	<.0001
Suicide attempt	≤10* (0.4)	≤10* (0.4)	1.000 (0.415‐2.407)	1
Acute Kidney Injury	60 (2.6)	199 (8.7)	0.283 (0.211‐0.380)	<.0001
Daytime sleepiness	197 (8.6)	102 (4.5)	2.019 (1.578‐2.583)	<.0001
Emergency department visit	344 (15.0)	639 (27.9)	0.457 (0.395‐0.529)	<.0001
Hospitalization	349 (15.2)	688 (30.0)	0.419 (0.362‐0.484)	<.0001
Aortic dissection	≤10* (0.4)	≤10* (0.4)	1.000 (0.415‐2.407)	1
Acute heart failure	19 (0.8)	93 (4.1)	0.198 (0.120‐0.325)	<.0001
Heart failure exacerbation	17 (0.7)	75 (3.3)	0.221 (0.130‐0.375)	<.0001
Acute respiratory failure	26 (1.1)	143 (6.2)	0.172 (0.113‐0.263)	<.0001
Pneumonia	45 (2.0)	167 (7.3)	0.255 (0.182‐0.356)	<.0001
Ventricular fibrillation	≤10* (0.4)	≤10* (0.4)	1.000 (0.415‐2.407)	1

Abbreviations: CI, confidence interval; CPAP, continuous positive airway pressure; HNS, hypoglossal nerve stimulation; OSA, obstructive sleep apnea.

*To protect patient privacy and maintain de‐identification, the TriNetX platform reports values of 10 or fewer as ≤10.

### HNS Versus UPPP Results

**Figure 3:**
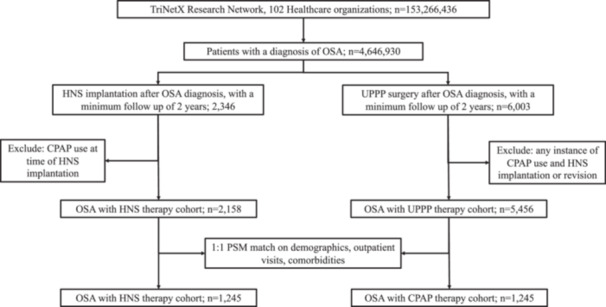
Flow diagram outlining inclusion, exclusion, and 1:1 propensity score matching of OSA patients receiving HNS versus UPPP therapy based on demographics, comorbidities, and outpatient visit frequency. HNS, hypoglossal nerve stimulation; OSA, obstructive sleep apnea; UPPP, uvulopalatopharyngoplasty.

A similar TriNetX cohort analysis was performed to compare patients who underwent HNS (n = 2158) versus UPPP (n = 5456) without subsequent CPAP use (Figure 3). Prior to PSM, there were significant differences observed between the cohorts, including sex, race/ethnicity, and multiple morbidities (SMD > 0.1) (Table 4). All comorbid conditions were more common in the HNS group, except for nicotine dependence, chronic sinusitis, tobacco use, respiratory failure, or acute ischemic heart disease, for which there were no differences between cohorts.

**Table 4:** 1:1 PSM of HNS and UPPP Cohorts

	Before PSM			After PSM		
Characteristic name	HNS (n = 2158); n (%)	UPPP (n = 5456); n (%)	SMD	HNS (n = 1245); n (%)	UPPP (n = 1245); n (%)	SMD
Age at index	61.9 ± 11.5	44.2 ± 13.0	**1.44**	56.6 ± 11.1	56.4 ± 10.6	0.017
White	1834 (85.0)	3524 (64.8)	**0.480**	994 (79.8)	995 (79.9)	0.002
Male	1402 (65.0)	3989 (73.3)	**0.181**	871 (70.0)	978 (70.6)	0.014
Female	715 (33.1)	1368 (25.1)	**0.177**	355 (28.5)	347 (27.9)	0.014
Black or African American	87 (4.0)	666 (12.2)	**0.304**	75 (6.0)	62 (5.0)	0.046
Hispanic or Latino	72 (3.3)	478 (9.0)	**0.235**	56 (4.5)	66 (5.3)	0.037
Asian	37 (1.7)	281 (5.2)	**0.190**	34 (2.7)	41 (3.3)	0.033
Hypertensive diseases	1222 (56.6)	1822 (33.5)	**0.478**	604 (48.5)	608 (48.8)	0.006
Disorders of lipoprotein metabolism and other lipidemias	1204 (55.8)	1618 (29.7)	**0.546**	584 (46.9)	604 (48.5)	0.032
Encounter for other special examination without complaint, suspected or reported diagnosis	1033 (47.9)	1976 (36.3)	**0.236**	542 (43.5)	537 (43.1)	0.008
BMI 30‐39, adult	519 (24.1)	528 (9.7)	**0.390**	241 (19.4)	242 (19.4)	0.002
Encounter for general examination without complaint, suspected or reported diagnosis	542 (25.1)	1022 (18.8)	**0.153**	274 (22.0)	262 (21.0)	0.024
Diabetes mellitus	459 (21.3)	631 (11.6)	**0.263**	207 (16.6)	209 (16.8)	0.004
Depression, unspecified	411 (19.0)	324 (6.0)	**0.404**	168 (13.5)	167 (13.4)	0.002
BMI 20‐29, adult	373 (17.3)	180 (3.3)	**0.473**	117 (9.4)	116 (9.3)	0.003
Chronic ischemic heart disease	352 (16.3)	285 (5.2)	**0.363**	139 (11.2)	139 (11.2)	<0.0001
Nicotine dependence	263 (12.2)	689 (12.7)	0.014	170 (13.7)	161 (12.9)	0.021
Chronic sinusitis	283 (13.1)	794 (14.6)	0.043	164 (13.2)	154 (12.4)	0.024
Atrial fibrillation and flutter	203 (9.4)	106 (1.9)	**0.327**	51 (4.1)	57 (4.6)	0.024
Other disorders of fluid, electrolyte and acid‐base balance	202 (0.4)	220 (4.0)	**0.214**	79 (6.3)	84 (6.7)	0.016
Chronic kidney disease	191 (8.9)	119 (2.2)	**0.300**	54 (4.3)	49 (3.9)	0.020
Other chronic obstructive pulmonary disease	145 (6.7)	159 (2.9)	**0.178**	64 (5.1)	58 (4.7)	0.022
Heart failure	115 (5.3)	109 (2.0)	**0.178**	38 (3.1)	47 (3.8)	0.040
Disorder of kidney and ureter, unspecified	104 (4.8)	87 (1.6)	**0.184**	31 (2.5)	38 (3.1)	0.034
Acute kidney failure	90 (4.2)	81 (1.5)	**0.162**	30 (2.4)	29 (2.3)	0.005
Tobacco use	77 (3.6)	121 (2.2)	0.080	43 (3.5)	41 (3.3)	0.009
Atherosclerosis	75 (3.5)	56 (1.0)	**0.165**	27 (2.2)	27 (2.2)	<0.0001
Cerebral infarction	78 (3.6)	56 (1.0)	**0.172**	28 (2.2)	31 (2.5)	0.016
Acute myocardial infarction	64 (3.0)	58 (1.1)	**0.136**	24 (1.9)	31 (2.5)	0.038
Respiratory failure, not elsewhere classified	46 (2.1)	55 (1.0)	0.090	13 (1.0)	19 (1.5)	0.043
Other pulmonary heart diseases	41 (1.9)	40 (0.7)	**0.102**	19 (1.5)	19 (1.5)	<0.0001
BMI 40 or greater, adult	21 (1.0)	237 (4.4)	**0.211**	19 (1.5)	11 (0.9)	0.059
Other acute ischemic heart diseases	14 (0.6)	10 (0.2)	0.072	10 (0.8)	10 (0.8)	<0.0001

Propensity score matching was done at a 1:1 ratio. Significant difference (bolded) was set at a standardized mean difference >0.10.

Abbreviations: HNS, hypoglossal nerve stimulation; PSM, Propensity score matching; SMD, standardized mean difference; UPPP, uvulopalatopharyngoplasty.

After matching (n = 1245 in both HNS and UPPP cohorts), patients who underwent HNS had lower odds of AKI (1.0% vs 2.0%, OR 0.52, 95% CI: 0.26‐1.01, *P* = .0498) and ED visit (10.9% vs 15.3%, OR 0.68, 95% CI: 0.54‐0.86, *P* = .001) (Table 5). The HNS cohort was more likely to experience daytime sleepiness (9.9% vs 7.0%, OR 1.50, 95% CI: 1.10‐1.94, *P* = .0094). All other measured adverse events occurred at similar rates in the 2 surgical cohorts.

**Table 5:** 30 Days to 2‐Year Outcomes of HNS Versus UPPP Therapy for OSA Patients

Outcome	HNS (n = 1245); n (%)	UPPP (n = 1245); n (%)	Odds ratio (95% CI)	*P*‐value
Stroke	17 (1.4)	13 (1.0)	1.312 (0.634‐2.713)	.4625
Myocardial infarction	13 (1.0)	15 (1.2)	0.865 (0.41‐1.826)	.7039
Atrial fibrillation/atrial flutter	41 (3.3)	42 (3.4)	0.975 (0.630‐1.511)	.9111
Hypertensive crisis	≤10* (0.8)	≤10* (0.8)	1.000 (0.415‐2.410)	1.0000
Pulmonary embolism	≤10* (0.8)	≤10* (0.8)	1.000 (0.415‐2.410)	1.0000
Ventricular tachycardia	≤10* (0.8)	≤10* (0.8)	1.000 (0.415‐2.410)	1.0000
COPD exacerbation	11 (0.9)	≤10* (0.8)	1.101 (0.466‐2.602)	.8265
Suicide attempt	≤10* (0.7)	0 (0.0)	‐	‐
Acute kidney injury	13 (1.0)	25 (2.0)	0.515 (0.262‐1.011)	**.0498**
Daytime sleepiness	123 (9.9)	87 (7.0)	1.459 (1.096‐1.943)	**.0094**
Emergency department visit	136 (10.9)	191 (15.3)	0.677 (0.535‐0.856)	**.0011**
Hospitalization	157 (12.6)	188 (15.1)	0.811 (0.646‐1.019)	.0721
Aortic dissection	0 (0)	≤10* (0.8)	‐	‐
Acute heart failure	≤10* (0.8)	≤10* (0.8)	1.000 (0.415‐2.410)	1.0000
Heart failure exacerbation	≤10* (0.8)	≤10* (0.8)	1.000 (0.415‐2.410)	1.0000
Acute respiratory failure	≤10* (0.8)	≤10* (0.8)	1.000 (0.415‐2.410)	1.0000
Pneumonia	26 (2.1)	20 (1.6)	1.306 (0.725‐2.353)	.3719
Ventricular fibrillation	0 (0)	0 (0)	‐	‐

Abbreviation: CI, confidence interval; HNS, hypoglossal nerve stimulation; OSA, obstructive sleep apnea; UPPP, uvulopalatopharyngoplasty.

*To protect patient privacy and maintain de‐identification, the TriNetX platform reports values of 10 or fewer as ≤10.

## Discussion

### HNS Versus CPAP

After matching for demographics and comorbidities, we found that HNS was associated with significantly lower risk of cardiovascular, respiratory, and renal outcomes, as well as decreased healthcare utilization, compared to CPAP therapy over a 2‐year window. One exception to this trend was excessive daytime sleepiness, which was more frequently documented in the HNS cohort.

A key driver of these findings may be differences in treatment adherence. CPAP adherence is recognized to be suboptimal in clinical practice.^23^, ^24^, ^25^ Despite limited data, it appears that HNS has better adherence especially with early use.^26^, ^27^ Another propensity matched study showed that patients have a significant increase in HNS of 2 to 3 hours more per night compared to CPAP over 3 months.^28^ Because adherence and therapeutic efficacy jointly determine clinical benefit, the disparity in outcomes may reflect differences in MDA between HNS and CPAP. A recent study showed upper airway surgery effectiveness in terms of MDA remained superior to CPAP.^19^ While adherence data is not available within TriNetX, our findings align with prior literature to support the importance of adherence in success of OSA clinical outcomes.

There were significantly higher incidence rates of acute cardiovascular outcomes observed in the CPAP group. These differences may be explained by inadequate OSA control in patients with poor CPAP compliance. Untreated or poorly controlled OSA has been strongly linked to increased risk of cardiovascular disease.^29^, ^30^, ^31^, ^32^ Without adequate compliance, CPAP may not mitigate these effects, allowing OSA mechanisms to persist, whereas higher HNS adherence may offer greater cardiovascular protection. Long‐term data analysis is needed to determine if these outcomes persist over time.

Interestingly, COPD exacerbation and pneumonia were significantly less common in the HNS cohort despite both groups being matched for baseline COPD prevalence. CPAP is protective and reduces COPD exacerbations and hospitalizations in patients with both OSA and COPD diagnosis (overlap syndrome).^33^ Therefore, the protective effects of HNS on respiratory function may actually be underestimated in this study. More information is needed to confirm these findings. One possible reason for increased pneumonia prevalence in the CPAP cohort may be due to improper hygiene of the CPAP machine which may increase infection risk.^34^ A significantly lower incidence of systemic findings reinforced the broad impact of HNS across organ systems for OSA patients, as the CPAP group demonstrated higher AKI rates.^35^, ^36^

Beyond clinical outcomes, HNS therapy was also associated with fewer ED visits and hospitalizations. These differences may reflect exacerbations or downstream complications of undertreated OSA, as well as the HNS population having fewer comorbidities.

An exception to this trend was daytime sleepiness, which was more commonly reported in the HNS cohort—possibly due to greater patient engagement, symptom awareness, and overall increased healthcare interaction from extensive pre‐ and post‐operative evaluation. Alternatively, a lack of immediate symptom relief may be more noticeable due to heightened expectations or ongoing device programming. Interestingly, a meta‐analysis found no difference in Epworth Sleepiness Scale (ESS) scores between HNS, CPAP, or UAS cohorts.^37^ However, this finding may suggest that CPAP and HNS may impact sleep quality differently, warranting further investigation of polysomnography objective findings.

It is also important to consider that patients undergoing HNS may differ in socioeconomic status (SES), access to subspecialty care, and health engagement, which could contribute to improved outcomes independent of the device itself. CPAP is relatively inexpensive and widely covered, whereas HNS is costly and requires stringent criteria, selecting for higher SES groups.^38^, ^39^ Studies have linked lower SES to greater OSA severity, reflected in AHI and oxygen desaturation index.^40^ Additionally, low SES is an independent risk factor for cardiovascular disease among OSA patients requiring treatment, further compounding health disparities.^41^

The finding of lower rates of several comorbidities, including heart failure, CKD, COPD, and diabetes, in the pre‐matched HNS group compared to the CPAP group may suggest that patients selected for HNS were healthier at baseline. This selection bias, even after rigorous propensity score matching, underscores the challenges of comparing different treatment modalities in observational studies. It is possible that clinicians preferentially offered HNS to a subset of OSA patients deemed more likely to benefit or tolerate the surgery. Thus, a second analysis was run comparing HNS to UPPP to determine if these differences seen in HNS vs CPAP cohorts were due to a healthier baseline surgical cohort.

### HNS Versus UPPP

Table 5 showed that HNS and UPPP had largely similar systemic outcomes, with the exceptions of subsequent AKI and ED visits being higher in UPPP patients. The degree of airway tissue trauma in UPPP is known to cause substantial pharyngeal pain, often requiring opioid analgesics for 7 to 14 days, with pain scores highest in the first postoperative week.^42^, ^43^, ^44^, ^45^ Therefore, initial postoperative UPPP pain alone may account for some of the observed differences in postoperative ED visits.

The extreme pain after UPPP surgery may also cause secondary poor oral intake and dehydration. In a large cross‐sectional study of 2349 ambulatory adult patients after UPPP, 6.6% patients had a revisit within 14 days due to fever or dehydration—the third most common reason after bleeding and acute pain.^46^ In contrast, HNS involves minimal dissection, without the violation of the oropharyngeal mucosa or the airway as seen in UPPP, allowing for quicker recovery in HNS compared to UPPP patients.

Postoperative renal outcomes, including AKI, are understudied in OSA surgery. While AKI is often attributed to intra‐ or postoperative hemorrhage, it is unlikely that intraoperative bleeding contributed in this study, since UPPP and HNS both involve minimal intraoperative blood loss. Although, UPPP carries some risk for postoperative hemorrhage compared to HNS.^46^, ^47^, ^48^ Post‐HNS hemorrhage is rare, and its incidence is not well described in the literature. It is possible that the increased AKI risk in the UPPP cohort partially arose from differences in postoperative dysphagia, leading to decreased oral intake and dehydration. Dysphagia after HNS surgery is minimal, and according to a single‐institution prospective study, minor difficulties with swallowing were temporary, resolving over 3 months.^49^ Conversely, up to 25% of patients experience temporary dysphagia after UPPP, and patients with pre‐existing swallowing disturbances may worsen outcomes after surgery.^50^, ^51^, ^52^ Difficulty swallowing may persist, which could potentially lead to future ED visits.

Beyond immediate postoperative effects such as pain and dysphagia, systemic outcomes were comparable between UPPP and HNS. This suggests that the observed advantages in the HNS versus CPAP analysis may, in part, reflect selection bias, as patients with newly diagnosed OSA and significant comorbidities are often not referred for surgery due to elevated perioperative risk. This may suggest that the HNS vs CPAP outcome differences may be due to factors other than adherence or treatment efficacy and more so to do with baseline population differences and how surgical candidacy represents better overall health. Nonetheless, differences in invasiveness, surgical technique, and patient selection between UPPP and HNS likely introduce confounding variables that limit direct comparability. Further research is warranted to delineate the population‐level benefits of traditional airway surgery relative to HNS.

This study has several limitations. First, TriNetX does not capture adherence data, OSA severity, or OSA therapy efficacy, as analyses are restricted to ICD‐10 and CPT codes. Because these factors are key determinants of long‐term outcomes in OSA, our methodology cannot determine whether observed differences reflect true disease alleviation vs differences in real‐world therapy use. The duration of OSA prior to treatment initiation was also unknown, which likely influenced both outcomes and downstream complications. Furthermore, variation in diagnostic and procedural coding across institutions may have introduced misclassification and heterogeneity. Although propensity matching mitigated baseline differences, residual confounding may persist, particularly if patients with greater disease burden were more likely to be managed with CPAP. Additionally, while the unmatched CPAP cohort may have worse baseline health, TriNetX does not provide data on disease severity or therapeutic effectiveness, limiting interpretation. As HNS eligibility requires CPAP failure, it is plausible that patients in the HNS cohort had a prolonged course of inadequately treated OSA. Although prior CPAP use is required, it was likely non‐therapeutic and not clinically effective. It is also possible that surgical candidates had socioeconomic advantages or better baseline health, potentially overestimating the protective effects of HNS. Finally, we were unable to account for variability in time to therapeutic stimulation in HNS patients or to distinguish whether outcome events, such as ED visits, occurred in the immediate postoperative period or later.

## Conclusion

In this large TriNetX study comparing HNS and CPAP therapy in OSA patients, HNS was associated with significantly lower risk of cardiovascular, renal, and respiratory outcomes, as well as reduced healthcare utilization over 2 years. Therefore, HNS may offer systemic and economic advantages over CPAP. However, these results may be at least partially attributed to a healthier surgery cohort. Given the limitations of administrative data and the inability to measure treatment adherence or OSA severity, future studies incorporating objective sleep metrics, device adherence, and cost‐effective analyses are needed to validate our findings.

## Author Contributions

**Iman Adibi**, manuscript writing, idea generation, manuscript editing, data analysis; **Amala Nayak**, manuscript writing, idea generation, manuscript editing, data analysis; **Arman Saeedi**, data analysis; **Aaron Tucker**, data analysis, manuscript editing; **Alyssa Calder**, manuscript editing; **Ryan Nord**: idea generation, manuscript editing, data analysis.

## Disclosures

### Competing interests

Ryan Nord: Paid consultant for Inspire Medical Systems, the manufacturer of the device studied in this research.

### Funding source

None.
